# Simultaneous Optimization of MP2RAGE T_1_ ‐weighted (UNI) and FLuid And White matter Suppression (FLAWS) brain images at 7T using Extended Phase Graph (EPG) Simulations

**DOI:** 10.1002/mrm.29479

**Published:** 2022-11-09

**Authors:** Ayşe Sıla Dokumacı, Fraser R. Aitken, Jan Sedlacik, Pip Bridgen, Raphael Tomi‐Tricot, Ronald Mooiweer, Katy Vecchiato, Tom Wilkinson, Chiara Casella, Sharon Giles, Joseph V. Hajnal, Shaihan J. Malik, Jonathan O'Muircheartaigh, David W. Carmichael

**Affiliations:** ^1^ Biomedical Engineering Department School of Biomedical Engineering and Imaging Sciences, King's College London London United Kingdom; ^2^ London Collaborative Ultra high field System (LoCUS) London United Kingdom; ^3^ Radiology Department Great Ormond Street Hospital for Children London United Kingdom; ^4^ MR Research Collaborations Siemens Healthcare Limited Camberley United Kingdom; ^5^ Department of Forensic and Neurodevelopmental Sciences Institute of Psychiatry, Psychology and Neuroscience, King's College London London United Kingdom; ^6^ Centre for the Developing Brain School of Biomedical Engineering and Imaging Sciences, King's College London London United Kingdom; ^7^ MRC Centre for Neurodevelopmental Disorders, King's College London London United Kingdom

**Keywords:** 7T, FLAWS, MP2RAGE, MRI, ultrahigh field

## Abstract

**Purpose:**

The MP2RAGE sequence is typically optimized for either T_1_‐weighted uniform image (UNI) or gray matter–dominant fluid and white matter suppression (FLAWS) contrast images. Here, the purpose was to optimize an MP2RAGE protocol at 7 Tesla to provide UNI and FLAWS images simultaneously in a clinically applicable acquisition time at <0.7 mm isotropic resolution.

**Methods:**

Using the extended phase graph formalism, the signal evolution of the MP2RAGE sequence was simulated incorporating T_2_ relaxation, diffusion, RF spoiling, and B_1_^+^ variability. Flip angles and TI were optimized at different TRs (TR_MP2RAGE_) to produce an optimal contrast‐to‐noise ratio for UNI and FLAWS images. Simulation results were validated by comparison to MP2RAGE brain scans of 5 healthy subjects, and a final protocol at TR_MP2RAGE_ = 4000 ms was applied in 19 subjects aged 8–62 years with and without epilepsy.

**Results:**

FLAWS contrast images could be obtained while maintaining >85% of the optimal UNI contrast‐to‐noise ratio. Using TI_1_/TI_2_/TR_MP2RAGE_ of 650/2280/4000 ms, 6/8 partial Fourier in the inner phase‐encoding direction, and GRAPPA factor = 4 in the other, images with 0.65 mm isotropic resolution were produced in <7.5 min. The contrast‐to‐noise ratio was around 20% smaller at TR_MP2RAGE_ = 4000 ms compared to that at TR_MP2RAGE_ = 5000 ms; however, the 20% shorter duration makes TR_MP2RAGE_ = 4000 ms a good candidate for clinical applications example, pediatrics.

**Conclusion:**

FLAWS and UNI images could be obtained in a single scan with 0.65 mm isotropic resolution, providing a set of high‐contrast images and full brain coverage in a clinically applicable scan time. Images with excellent anatomical detail were demonstrated over a wide age range using the optimized parameter set.

## 1 INTRODUCTION

The MP2RAGE sequence^1^ is an extension of the MPRAGE sequence^2^ and it is commonly used for 3D structural T_1_‐weighted imaging of the brain at 7 Tesla (T). This sequence acquires 2 rapid gradient echo (GRE) images (INV1 and INV2) at 2 TIs after the application of a nonselective 180° adiabatic inversion pulse.^1^ These images are combined via a complex ratio to produce a more uniform T_1_‐weighted image (UNI) compared to that produced with the MPRAGE sequence. The UNI image is insensitive to receive field (B_1_^−^) inhomogeneity, proton density, and T_2_* effects^1^, ^3^; however, it is still sensitive to inhomogeneity in the transmit field (B_1_^+^), resulting in regional variation in contrast and SNR.

The MP2RAGE sequence is typically optimized to obtain UNI images with maximum contrast‐to‐noise ratio (CNR) between white matter (WM), gray matter (GM), and CSF.^1^ Due to the limited use of INV1 and INV2 images in clinical applications,^1^ the MP2RAGE sequence has also been optimized to produce an INV1 image with WM suppression and an INV2 image with CSF suppression at 3T.^4^ The image with WM suppression was similar to a fast gray matter acquisition T_1_ inversion recovery image^5^ that would be beneficial for the visualization of the subcortical brain structures.^4^ The image with CSF suppression would be used for structural imaging^4^; however, it would not be purely T_1_‐weighted. By taking the minimum intensity projection of these INV1 and INV2 images, fluid and white matter suppression (FLAWS) images with similar contrast to double inversion recovery images^6^, ^7^ have been produced and have shown potential utility for clinical applications, including the improved detection of epileptogenic zones in focal cortical dysplasia.^4^, ^8^ In addition, the acquisition of 2 images with different contrasts within 1 scan eliminated the need for coregistration.^4^ The MP2RAGE sequence optimized for FLAWS images has also been shown to outperform the double inversion recovery turbo spin echo sequence in healthy subjects at 7T in terms of WM suppression, GM delineation, and SAR, whereas the double inversion recovery turbo spin echo sequence was shown to be better at suppressing CSF.^9^

Beaumont et al. optimized the FLAWS images for deep brain stimulation surgery planning at 1.5 T and derived a new set of image combinations called FLAWS_hc_ (hc: high‐contrast) and FLAWS_hco_ (hco: opposite contrast to hc).^10^ Like the MP2RAGE UNI image, the FLAWS_hc_ and FLAWS_hco_ images are obtained using a ratio of the INV1 and INV2 images and are therefore insensitive to B_1_^−^ inhomogeneity, proton density, and T_2_* effects.^10^ Recently, the FLAWS images were optimized for 7T using Bloch simulations.^11^ The resultant MP2RAGE UNI image had GM suppression instead of a typical T_1_‐weighted contrast, which required the use of FLAWS_hco_ images for T_1_‐weighted contrast. Because the FLAWS_hc_ and FLAWS_hco_ images are also affected by B_1_^+^ variability, the effects of the B_1_^+^ should be considered for optimizations at ultrahigh field strengths.^10^ MP2RAGE is an important sequence shown to be effective at 7T for epilepsy diagnosis, in part owing to the various contrasts it can generate from a single sequence.^12^

The aim of this study was to obtain an optimized MP2RAGE imaging protocol for use at 7T to provide both FLAWS and T_1_‐weighted UNI images within a clinically applicable single scan at <0.7 mm isotropic resolution. For this purpose, shorter than typical MP2RAGE TRs (TR_MP2RAGE_ = 4000 ms) and acquisition times were investigated to allow for faster imaging. Employing the extended phase graph (EPG) formalism, which characterizes the magnetization response of a large range of MR sequences efficiently in the Fourier domain,^13^, ^14^, ^15^ we incorporated previously neglected potential effects from T_2_ relaxation, RF spoiling performance, and the B_1_^+^ variability encountered at 7T. Using optimized scan parameters derived from these simulations, we were able to demonstrate that high‐quality FLAWS and UNI images could be obtained in vivo across a wide age range with 0.65 mm isotropic resolution in under 7.5 min with full brain coverage. The results of this study were presented in part as abstracts.^16^, ^17^

## 2 METHODS

### 2.1 EPG simulations

An EPG simulation for GRE sequences^18^, ^19^ was modified for MP2RAGE and performed in MATLAB (R2018a, MathWorks, Natick, MA). In the MP2RAGE sequence,^1^ after the application of a nonselective adiabatic inversion pulse, the first GRE image (INV1) is acquired. The time between the inversion pulse and the acquisition of the center of k‐space for the first GRE block is defined as the first TI (TI_1_). Similarly, the second GRE image (INV2) is acquired in the second GRE block, and the second TI (TI_2_) is defined with respect to the same shared inversion pulse. The TR of the MP2RAGE sequence (TR_MP2RAGE_) is defined as the time between 2 consecutive inversion pulses. TR_GRE_ is the time between each excitation in the GRE blocks. In the sequence implementation, the RF phase spoiling had a quadratic cycling pattern and a phase increment of 50°, which were included in the simulations. No assumption was made for the complete spoiling of the transverse magnetization. Diffusion effects were also incorporated.^15^, ^18^ Literature adult T_1_/T_2_ values of 1220/45.9, 2132/55, and 3350/1000 ms and proton density values of 0.69, 0.82, and 1 were used for WM, GM, and CSF, respectively.^1^, ^20^, ^21^

The large B_1_^+^ variation at 7T^1^ was considered in the simulations by applying a range of scaling factors (0.5–1.4 with steps of 0.1) to the nominal flip angles (FAs) to achieve a B_1_^+^ range of 50%–140% with steps of 10%.

### 2.2 Optimization of TIs at different TR_MP2RAGE_ values

Beaumont et al.^11^ used TR_MP2RAGE_ = 5000 ms for an MP2RAGE sequence to obtain FLAWS images at 7T. In this study, 3 different TR_MP2RAGE_ values were tested to allow for faster acquisitions: 4000, 4500, and 5000 ms. In the original MP2RAGE study by Marques et al.,^1^ TR_GRE_ = 7.2 ms was used for high‐resolution (0.5–0.8 mm^3^) images. Here, a similar value (TR_GRE_ = 7.26 ms) with a resolution <0.7 mm was used. Partial Fourier acquisitions of 6/8 were employed in both phase‐encoding directions in this first set of simulations to enable the greatest range of plausible TIs (TI_1_ and TI_2_) to be explored. One hundred and eighty excitations per GRE block resulted from 240 steps in the first phase‐encoding (partition) direction (acquired in the innermost k‐space loop).

To optimize the choice of TIs, the MP2RAGE signals for different tissue types were simulated using different combinations of GRE FAs and TIs at these different TR_MP2RAGE_ values. The TI_1_ values starting from 600 ms up to a maximum value of 1600 ms (depending on the TR_MP2RAGE_), with steps of 200 ms; and TI_2_ values starting from 2000 ms up to a maximum value of 3200 ms, with steps of 200 ms, were used. The FA range was 1–9° for the first FA (*α*_1_) and 1–17° for the second FA (*α*_2_), both with steps of 2°. Several further simulations were performed using an FA range of 1–10° for both *α*_1_ and *α*_2_ with finer increments of 1° to investigate shorter TI_1_s, such as those using TI_1_s of 500–650 ms with 50 ms increments at a fixed TI_2_ of 2200 ms at TR_MP2RAGE_ = 4500 ms.

Independent Gaussian noise was added 100,000 times to the signal from the center k‐space point at each TI.^1^, ^22^ The noise was added at a constant level (for the same TR_GRE_) to have an SNR of approximately 20 in the INV2 image. Simulations considering 10 different B_1_^+^ values covering the range of B_1_^+^ values expected at 7T were performed, which led to a 3D matrix of size 10 × 100,000 × the number of FA combinations for each tissue type after the addition of noise for 100,000 times. For each FA combination, the CNR between two tissue types was calculated using the mean of the difference matrix between the signal intensities of the tissues divided by its SD.

The FLAWS_min_ signal is calculated as the minimum between the INV1 (suppressed WM) and INV2 (suppressed CSF) signals normalized by their sum.^4^ For the optimization of FLAWS_min_ and UNI contrasts simultaneously, the total CNR was defined as the sum of GM‐CSF and GM‐WM CNRs for FLAWS_min_ for a dominant GM signal and WM–GM and GM–CSF CNRs for UNI, which can be expressed as CNR_total_ = FLAWS_min_ CNR_GM‐CSF_ + FLAWS_min_ CNR_GM‐WM_ + UNI CNR_WM‐GM_ + UNI CNR_GM‐CSF_. All CNR_total_ values were divided by the square root of TR_MP2RAGE_ (in ms) and are given as CNRs per unit time. They were plotted utilizing the *contour* function in MATLAB (R2018a, MathWorks, Natick, MA) with 8 levels.

### 2.3 Optimization of the FAs

After deciding on the TI_1_/TI_2_ combination, the next step was the FA optimization. The target was to maximize the GM–CSF and GM–WM CNRs in the FLAWS_min_ image for a GM–dominant contrast while maintaining 85% of the maximum total UNI CNR (UNI CNR_WM‐GM_ + UNI CNR_GM‐CSF_). In other words, the best possible FLAWS_min_ image was determined without compromising the total UNI CNR by more than 15%.

At all TR_MP2RAGE_ values, simulations using the same TI_1_/TI_2_ values of 650/2220 ms were performed, leading to initial protocols for the in vivo scans summarized in Table 1. A set of simulations was performed at the shortest TR_MP2RAGE_ value (TR_MP2RAGE_ = 4000 ms) to optimize the FAs for a protocol using TR_GRE_ = 7.9 ms and TI_1_/TI_2_ = 650/2280 ms, with partial Fourier (6/8) only in the first phase‐encoding direction in order to obtain a short TI_1_, necessary for WM suppression in the INV1 image.^4^ This avoids the loss in image resolution associated with using partial Fourier acquisitions in multiple encoding directions. An FA range of 1–10° was considered with finer steps of 1° for both *α*_1_ and *α*_2_ independently.

**TABLE 1:** In vivo scan parameters for the initial protocols with the optimum FA combinations for a chosen TI_1_/TI_2_ combination at different TR_MP2RAGE_s. The first set was used to establish the validity of the simulations by intentionally choosing *α*_2_ to result in poor CNR and contrast reversal

TR_MP2RAGE_ (ms)	*α*_1_ (°)	*α*_2_ (°)	TI_1_ (ms)	TI_2_ (ms)	Scan duration (min:s)
4000	5	2	650	2220	7:10
4000	5	4	650	2220	7:10
4500	5	6	650	2220	8:03
5000	5	7	650	2220	8:57

Abbreviations: FA, flip angle.

To investigate the sensitivity of T_1_ variations on the optimum FA choice, the simulations were repeated for a range of T_1_ values (16 different sets of T_1_s) for WM (800–1150 ms) and GM (1550–1900 ms) at TR_MP2RAGE_ = 4000 ms.

For the protocol using TR_GRE_/TR_MP2RAGE_ = 7.9/4000 ms, the effect of the inversion pulse efficiency (*eff*) in the simulations was investigated using *eff* = 0.96, the value previously determined via numerical simulations by Marques et al.^1^ For all other simulations in this study, *eff* = 1 was assumed for the hyperbolic secant adiabatic inversion pulse.^23^, ^24^ Additionally, MP2RAGE UNI signals^1^ for WM/GM/CSF were simulated for this protocol to observe the effects of diffusion, T_2_ relaxation, and RF phase cycling (off/on) using a 100% B_1_^+^.

### 2.4 In vivo scans

To validate the simulation results, in vivo images with 0.65 mm isotropic resolution were acquired with the parameters summarized in Table 1 at different TR_MP2RAGE_ values in 4 healthy subjects using a Magnetom Terra (Siemens Healthcare, Erlangen, Germany) 7T system. A suboptimal protocol at TR_MP2RAGE_ = 4000 ms using an FA combination of 4/2° was also applied to establish the validity of the simulation results. Bloch simulations were performed to determine the inversion efficiency in these 4 subjects. For each subject, average *eff* and its SD over the 3D volume were calculated. A fifth healthy subject was scanned at TR_MP2RAGE_ = 4000 ms using several different FA combinations. Parallel imaging was employed with a GRAPPA^25^ acceleration factor of 3 and 40 autocalibration signal lines in the second phase‐encoding direction (outer loop). Written consent was obtained from all subjects (age: 31 ± 2 years, 4 m/1f). A 1TX/32‐channel RX coil was used (Nova Medical, Wilmington, MA).

Two different bandwidths (BW) of 160 Hz/Px and 350 Hz/Px resulting in TR_GRE_ values of 7.9 and 5.2 ms, respectively, were compared in 1 subject. For the scans with BW = 350 Hz/Px, a partial Fourier factor of 6/8 was used only in the second phase‐encoding direction (outer loop), with a GRAPPA factor of 3 in the partition direction (inner loop). For the scan with BW = 160 Hz/Px, the same partial Fourier factor was applied only in the partition direction (inner loop), with a GRAPPA factor of 4 in the outer loop to match the acquisition durations (7:10 vs. 7:18 min:s). The data were acquired using TR_MP2RAGE_ = 4000 ms with similar TIs (TI_1_/TI_2_ = 650/2220 ms vs. TI_1_/TI_2_ = 650/2280 ms) at a nominal resolution of 0.65 × 0.65 × 0.65 mm^3^. For the scans with BW = 350 Hz/Px, *α*_1_/*α*_2_ = 3/3° and *α*_1_/*α*_2_ = 3/4° were applied, with the latter expected to provide a better GM–CSF contrast. For the scan with BW = 160 Hz/Px, *α*_1_/*α*_2_ = 4/4° was employed. These FA combinations were chosen following the optimization procedure described previously.

To observe the effect of B_1_^+^ on contrast, simulations were performed using 50%/100%/150% B_1_^+^ for 3 different protocols given in Table 2: our final protocol with 0.65 mm isotropic resolution using TR_MP2RAGE_ = 4000 ms and partial Fourier acquisition in only 1 phase‐encoding direction, the MP2RAGE protocol optimized for low B_1_^+^ sensitivity by Marques et al.,^1^ and the FLAWS protocol by Beaumont et al.^11^ All protocols were applied in 1 subject. A GRAPPA factor of 4 was used for our final protocol, whereas 3 was applied for the others.

**TABLE 2:** Final protocol: suggested scan parameters for optimal combined UNI and FLAWS protocol at 7T that would be suitable for a broad age range from this study, low‐B_1_^+^‐sensitive MP2RAGE,^1^ and FLAWS protocols^11^

Protocol name	Final protocol	Low‐B_1_^+^‐sensitive MP2RAGE^1^	FLAWS^11^
TR_MP2RAGE_/TR_GRE_/TE (ms)	4000/7.9/3.15	8000/6.9/3.07	5000/5/2.06
TI_1_/TI_2_ (ms)	650/2280	1000/3300	620/1430
*α*_1_/*α*_2_	4°/5°	4°/5°	4°/8°
FOV (mm^2^)	208 × 208	208 × 208	240 × 240
Slices per slab	256	160	192
Acquisition matrix	320 × 320	208 × 208	300 × 300
Resolution (mm^3^)	0.65 × 0.65 × 0.65	1 × 1 × 1	0.8 × 0.8 × 0.8
Slice partial Fourier	6/8	6/8	6/8
Bandwidth (Hz/Px)	160	280	370
GRAPPA factor	4 (or 3)	3	3
Scan duration (min:s)	7:18 (or 8:58)	12:50	10:02

*Note*: For the comparison of the protocols, a GRAPPA factor of 4 was used for the final protocol.

Abbreviations: FLAWS, fluid and white matter suppression; T, tesla; UNI, uniform image.

Using the final protocol, a sample of 19 subjects with and without epilepsy aged 8–62 years was scanned. We recruited families for a prospective study of pediatric epilepsy (ethics reference 18/LO/1766). Informed consent was obtained from all participants or their legal representatives, as appropriate. All healthy adult scans were performed according to the local ethics approval (HR‐18‐19‐8700). Datasets from 2 pediatric patients were rejected owing to significant motion artifacts. A GRAPPA acceleration factor of 4 was chosen for the pediatric participants to reduce the scan time (the total scan duration was 7:18 min:s); however, a factor of 3 can be applied in compliant subjects in 8:58 min:s.

### 2.5 Image analysis

Image processing was performed using MATLAB R2018a (MathWorks). The DICOM images were converted to NIfTI using the dicm2nii function,^26^ and all images at different TR_MP2RAGE_ values including the FLAWS‐related images were coregistered and segmented (*P* > 0.99 to avoid including voxels with tissue partial volumes) using the SPM12^27^ software package. FLAWS_hco_ image at TR_MP2RAGE_ = 5000 ms was chosen as the reference image for registration and segmentation. The CNRs for each TR_MP2RAGE_ were estimated from the difference between the mean tissue signal intensities divided by the median absolute deviation of the UNI WM signal. To observe the contrast changes using different FA combinations at TR_MP2RAGE_ = 4000 ms, the ratios of the signal differences between 2 different tissues to the UNI WM signal intensities were calculated. These were WM–GM and GM–CSF for the UNI (T_1_‐weighted) image and GM–WM and GM–CSF for the FLAWS_min_ to obtain a GM‐dominant image.

The main image derived with a complex division from the MP2RAGE INV1 and INV2 images is the UNI.^1^ In order to produce the additional FLAWS contrasts from the INV1 and INV2 images, the images were first scaled to a common range. FLAWS_min_ images were obtained using the minimum intensity projection of the INV1 and INV2 magnitude images.^4^ FLAWS_hco_ images were calculated using the expression (INV2 ‐ INV1)/(INV1 + INV2), yielding images with similar contrast to the UNI image.^10^ Furthermore, FLAWS_hc_ images were produced with opposite contrast to the FLAWS_hco_ images.^10^ Images were produced offline; however, these simple ratios can be generated on the scanner similarly to the current MP2RAGE UNI images.

The UNI and FLAWS‐related images from a similar anatomical location and bias fields produced using SPM12^27^ were compared for the protocols summarized in Table 2. “Check Reg” function of SPM12^27^ was used to choose 3 areas in the UNI images with different B_1_^+^ values.

For the images acquired using the final protocol, the enhanced background noise owing to image division was removed using the method by O'Brien et al.^28^ For this purpose, the expression for FLAWS_hco_ was modified as (INV2 ‐ INV1 ‐ β)/(INV1 + INV2 + 2β), where *β* is an empirical parameter set to 70. The same *β* parameter was used for the FLAWS_hc_ image. FLAWS_min_ image was obtained as the minimum intensity projection of the FLAWS_hc_ and FLAWS_hco_ images. This was the same as the minimum intensity projection of INV1 and INV2 magnitude images, with the advantage of background noise correction.

## 3 RESULTS

### 3.1 Optimization of the TIs at different TR_MP2RAGE_ values

**FIGURE 1:**
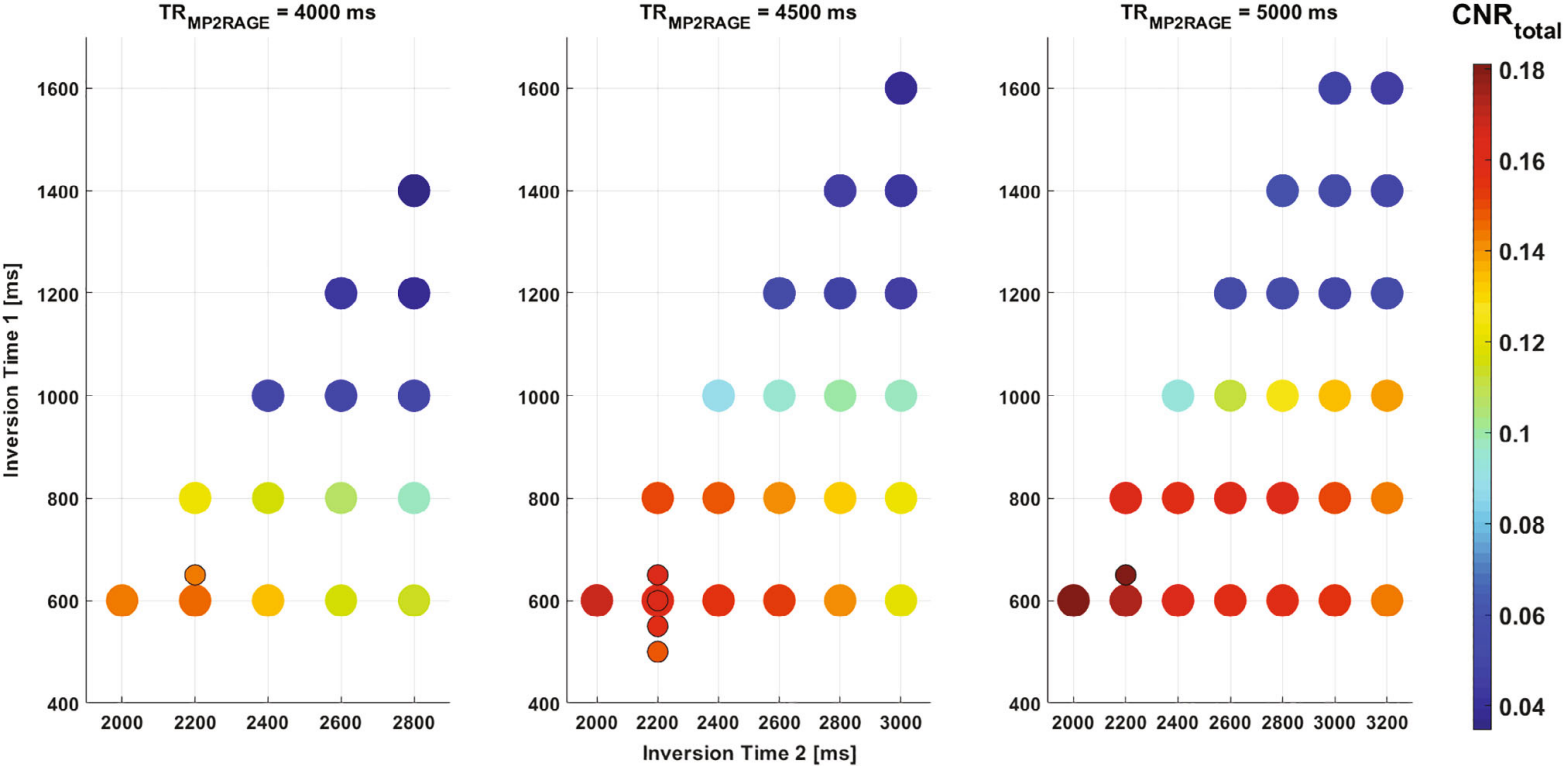
CNR_total_ (taking into account the CNRs of UNI and FLAWS_min_) obtained from EPG simulations using different TI_1_ and TI_2_ values for 3 choices of TR_MP2RAGE_. Each CNR_total_ value represented by a colored disk is the maximum found over the range of FAs tested, where for each FA the CNR is the average using all B_1_^+^ scaling values.  CNR, contrast‐to‐noise ratio; EPG, extended phase graph; FA, flip angle; FLAWS, fluid and white matter suppression; FLAWS_m__i__n_, FLAWS minimum image; UNI, uniform image.

Figure 1 shows the maximum CNR_total_ for the UNI and FLAWS_min_ (out of 45 FA combinations: 5 values for *α*_1_ and 9 for *α*_2_) for each TI_1_/TI_2_/TR_MP2RAGE_ represented by colored disks. Additional simulations, which more densely sampled TI_1_ times and FAs, are shown with smaller disks. In general, across different TR_MP2RAGE_ values, shorter TI_1_ times resulted in higher CNRs. At all TR_MP2RAGE_s, TI_1_/TI_2_ combinations of 600/2000 ms, 600/2200 ms, and 650/2200 ms provided very similar maximum CNRs (within 2.6%). Based on these results, a TI_1_/TI_2_ combination of 650/2220 ms was chosen for all protocols at all TR_MP2RAGE_s, being the closest achievable parameter combination on the scanner. According to the simulations, the CNR_total_ for UNI and FLAWS_min_ using TI_1_/TI_2_ of 650/2200 ms would be 0.1442, 0.1650, and 0.1797 at TR_MP2RAGE_ = 4000 ms, TR_MP2RAGE_ = 4500 ms, and TR_MP2RAGE_ = 5000 ms, respectively.

**FIGURE 2:**
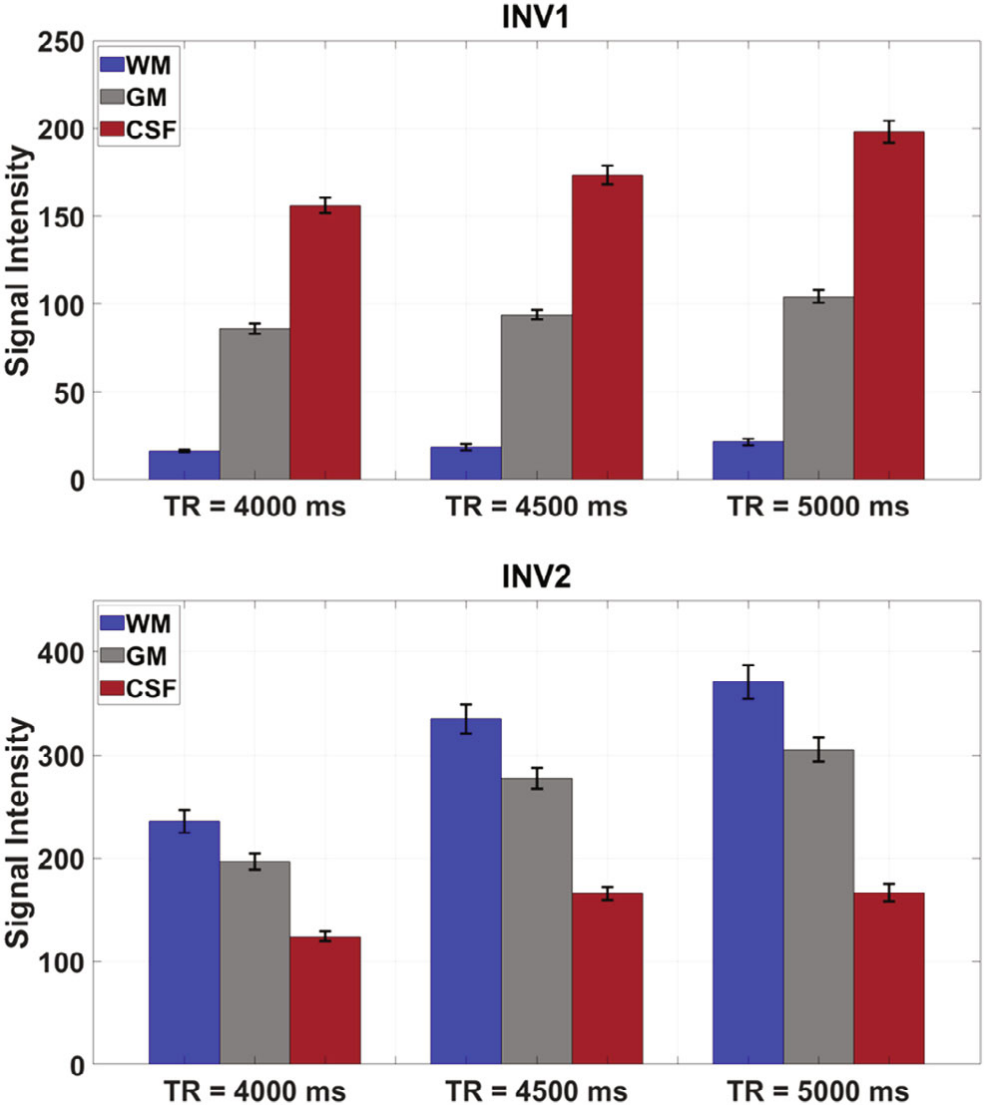
The INV1 and INV2 mean signal intensities at different TR_MP2RAGE_s for different tissues with the uncertainty of the mean, indicated as the error bars using the 3D segmented data from 4 healthy adults

To validate the simulation results above, 4 healthy adults were scanned with consistent parameters. Figure 2 demonstrates the results from these in vivo data across different TR_MP2RAGE_ values to achieve FLAWS type contrasts (WM and CSF signal suppression in the INV1 and INV2 images, respectively). The mean signal intensities for different tissues with the uncertainty of the mean indicated as the error bars from the 3D segmented data of 4 healthy adults using the initial protocols (Table 1) are given. WM suppression in the INV1 images was achieved for all subjects using different TR_MP2RAGE_s. In addition, significantly lower CSF signal intensities compared to the WM and GM signal intensities were achieved for all INV2 images. Table 3 summarizes the CNRs between WM–GM and GM–CSF in the UNI images calculated for each TR_MP2RAGE_. In all subjects, the UNI WM–GM CNR was lower at TR_MP2RAGE_ = 4000 ms compared to those at longer TR_MP2RAGE_s; however, the images still had high CNR. Moreover, the UNI GM‐CSF CNR was significantly lower at TR_MP2RAGE_ = 4000 ms using this FA combination (*α*_1_/*α*_2_ = 5°/4°) compared to those at longer TR_MP2RAGE_s. These were in line with the simulation results.

**FIGURE 3:**
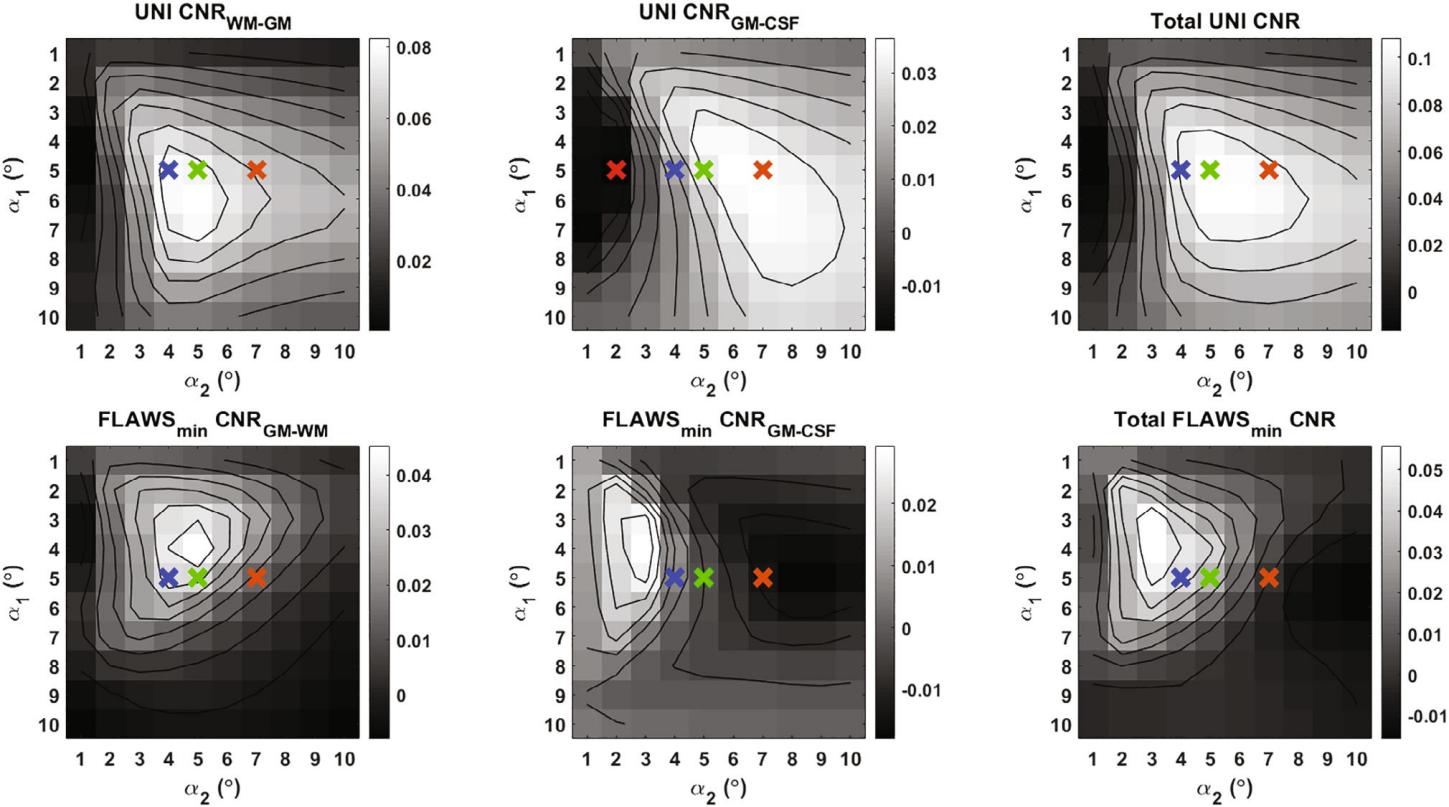
The UNI and FLAWS_min_ CNR plots using different FA combinations at a fixed TI_1_/TI_2_ of 650/2220 ms and TR_MP2RAGE_ of 4000 ms. The plots represent the average of simulation results using 10 different B_1_^+^ values (50% B_1_^+^ to 140% B_1_^+^ with steps of 10%). The plots considering individual B_1_^+^ values are given in Supporting Information Video S1. The upper row shows the CNRs for the UNI image between WM–GM and GM–CSF, and the total CNR. The lower row shows the CNRs for the FLAWS_min_ image between the GM–WM and GM–CSF, and the total CNR. The crosses correspond to different FA combinations (red: 5°/2°, blue: 5°/4°, green: 5°/5°, orange: 5°/7°). CNR, contrast‐to‐noise ratio; CS, compressed sensing; GM, gray matter; WM, white matter

**TABLE 3:** The UNI image CNRs from the data of 4 healthy adults and simulations at different TR_MP2RAGE_s using TI_1_/TI_2_ of 650/2220 ms

		TR_MP2RAGE_ = 4000 ms	TR_MP2RAGE_ = 4500 ms	TR_MP2RAGE_ = 5000 ms
UNI WM‐GM	Subject 1	7.897 (83.6%)	9.002 (95.3%)	9.446 (100%)
	Subject 2	7.948 (82.3%)	8.612 (89.2%)	9.655 (100%)
	Subject 3	8.538 (89.6%)	9.633 (101%)	9.530 (100%)
	Subject 4	7.918 (79.3%)	9.099 (91.2%)	9.979 (100%)
	Simulations	0.080 (88.0%)	0.085 (93.6%)	0.091 (100%)
UNI GM‐CSF	Subject 1	3.104 (47.8%)	6.437 (99.2%)	6.490 (100%)
	Subject 2	2.182 (41.8%)	4.991 (95.7%)	5.215 (100%)
	Subject 3	2.598 (46.9%)	5.810 (105%)	5.534 (100%)
	Subject 4	2.302 (42.6%)	5.283 (97.8%)	5.401 (100%)
	Simulations	0.018 (54.3%)	0.032 (99.7%)	0.032 (100%)

*Note*: For each row, the values in parenthesis show the relative CNR with respect to the CNR at TR_MP2RAGE_ = 5000 ms, which is assigned a value of 100%.

Abbreviations: CNR, contrast‐to‐noise ratio; GM, gray matter; WM, white matter.

### 3.2 FA optimization

**FIGURE 4:**
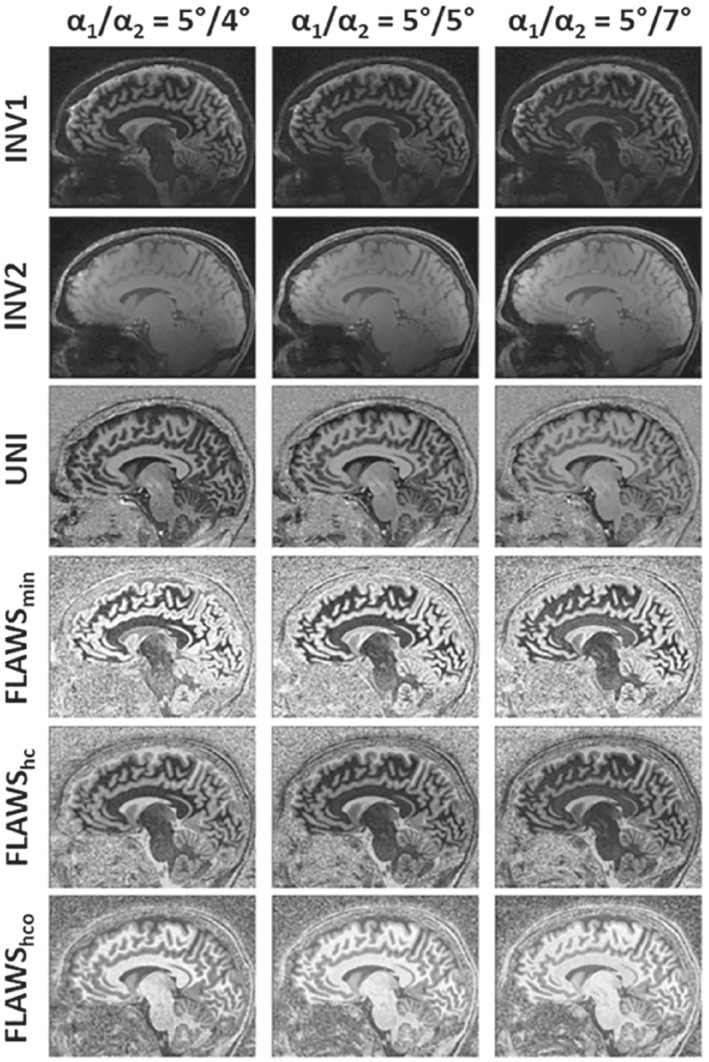
Various contrasts obtained from subject 5 at TR_MP2RAGE_ = 4000 ms using different *α*_2_ values

The CNR values obtained from EPG simulations of the MP2RAGE sequence using a range of FA combinations are given in Figure 3. The plots indicate that by modifying only *α*_2_ from 4° (blue cross) to 2° (red cross), the GM–CSF contrast would be reversed in the UNI image (Supporting Information Figure S1). There is a tradeoff between the UNI GM‐CSF and FLAWS_min_ GM–CSF contrasts. The blue, green, and orange crosses represent the *α*_1_/*α*_2_ combinations of 5°/4°, 5°/5°, and 5°/7°, respectively. They are all in the top 15% for the total UNI CNR and represent parameter combinations where only *α*_2_ is different. Table 4 shows the contrast changes due to different α_2_ values for the fifth healthy subject. As *α*_2_ increases, the GM–CSF contrast increases in the UNI and decreases in the FLAWS_min_ images, validating the simulation results. Figure 4 shows the different types of images from this subject, including the UNI and FLAWS_min_ where the GM–CSF contrast tradeoff can be observed. In addition, the FLAWS_hco_ images can be complementary to the UNI images by improving the GM–CSF contrast even if the contrast is suboptimal in the UNI image.

**TABLE 4:** The contrasts between two tissue types represented as percentages of the UNI WM signal calculated from the UNI and FLAWS_min_ images of subject 5 by using different second FAs

*α*_1_/*α*_2_	5/4° (blue)	5/5° (green)	5/7° (orange)
UNI GM‐CSF	22.82 ± 0.03	37.39 ± 0.03	46.12 ± 0.02
FLAWS_min_ GM‐CSF	0.63 ± 0.05	0.12 ± 0.05	0.00 ± 0.05
UNI WM‐GM	61.26 ± 0.03	52.05 ± 0.02	38.61 ± 0.02
FLAWS_min_ GM‐WM	126.06 ± 0.03	115.86 ± 0.03	100.52 ± 0.03

*Note*: TI_1_/TI_2_ of 650/2220 ms and TR_MP2RAGE_ of 4000 ms were used.

The results in Figure 3 were obtained from the simulations using the fixed T_1_ values from literature. It was observed that different T_1_ values for WM and GM could change the FAs required to obtain the desired combination of CNR values. At TR_MP2RAGE_ = 4000 ms, the *α*_1_ and *α*_2_ values for maximum CNR_total_ were found to be 4.1° ± 0.5° and 3.1° ± 0.6° (where the errors are the SDs of the optimum values using 16 different sets of T_1_s), respectively, whereas using *α*_1_ = 5.1° ± 0.3° and *α*_2_ = 5.2° ± 1.3° would lead to maximum total UNI CNR over the range of T_1_ values tested.

### 3.3 In vivo scans

The average *eff* was very close to 1 (0.99 over 4 subjects). In 1 subject, a small set of voxels in the cerebellum had lower B_1_^+^ and substantially reduced *eff*. The simulated UNI signals^1^ for WM/GM/CSF using *eff* = 0.96 and *eff* = 1 are given in Supporting Information Figure S2, where only a subtle difference can be observed. The effects of diffusion, T_2_ relaxation, and RF spoiling on the UNI signal intensities are given in Supporting Information Figure S3. For all simulated tissues (WM/GM/CSF), if the T_2_ effects were excluded, neither RF spoiling nor diffusion significantly altered the simulated signals (the curves generated using 4 different RF spoiling and diffusion combinations were overlapping). When including T_2_ relaxation with RF spoiling and diffusion (as used in all other simulations of this study), very similar results were obtained but with additional small signal oscillations. As expected, including T_2_ effects had the greatest impact on CSF signal simulations due to its significantly longer T_2_ value, including stronger changes when modeling diffusion and RF spoiling.

Supporting Information Figure S4 shows UNI images acquired using 2 different bandwidths. The images acquired with BW = 160 Hz/Px had higher SNR (visual inspection only) and no visually apparent chemical shift artifacts.

**FIGURE 5:**
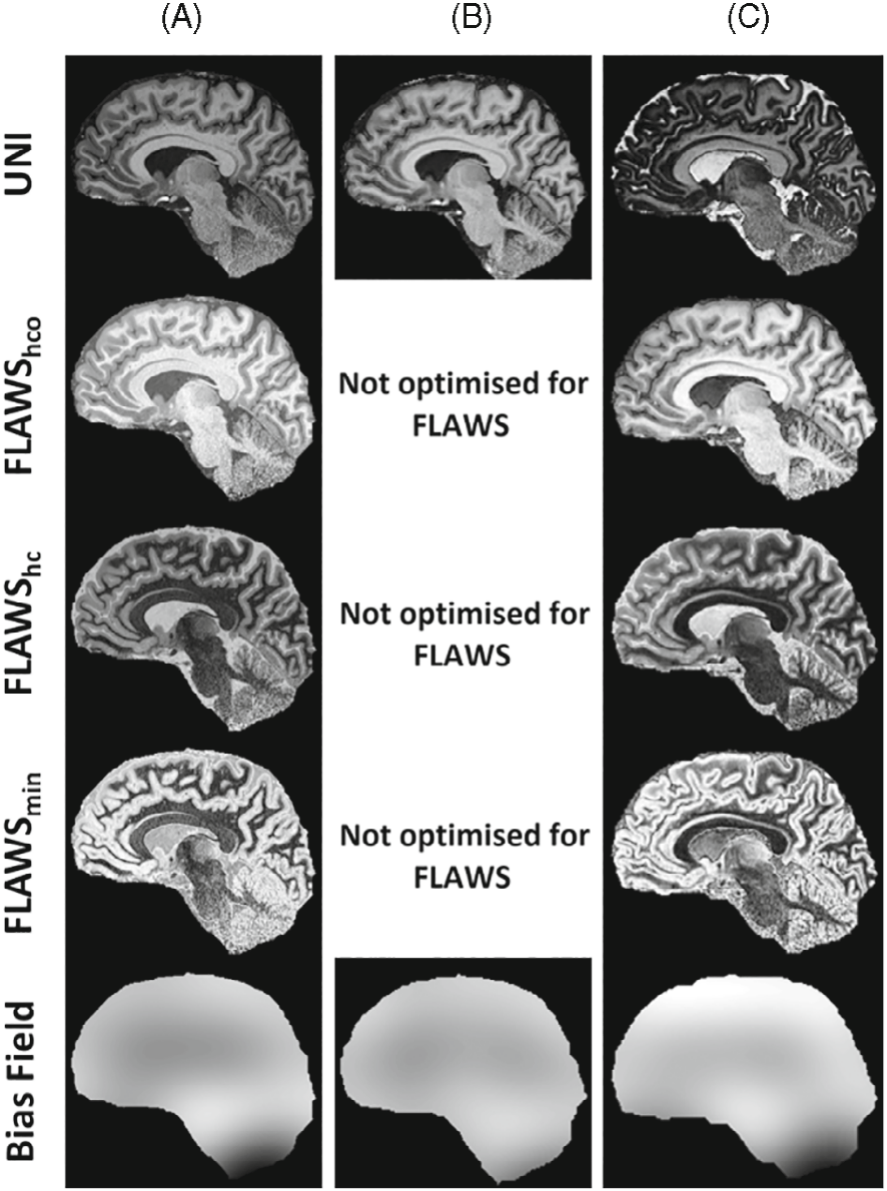
The UNI and FLAWS‐related images and the bias fields calculated from the UNI images using (A) the final protocol (B) the MP2RAGE protocol with low sensitivity to B_1_^+^ by Marques et al.^1^ (C) the protocol optimized for FLAWS contrast by Beaumont et al.^11^ The details of the protocols are given in Table 2. It is important to note that the final protocol has a shorter TR_MP2RAGE_ and scan duration and a higher nominal resolution compared to those of the other 2 protocols

**FIGURE 6:**
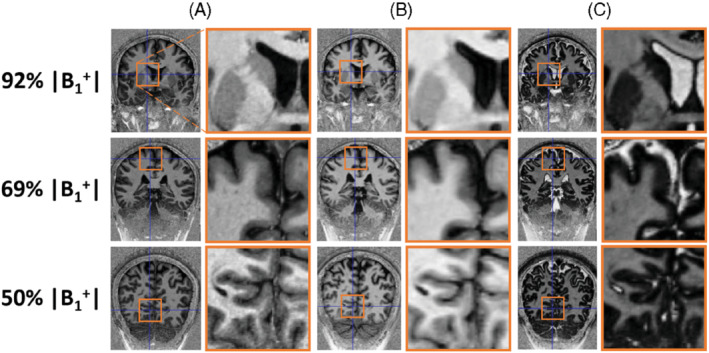
Three areas (each 40 × 40 mm^2^) having different B_1_^+^ values selected from the UNI images that were acquired using (A) the final protocol (B) the MP2RAGE protocol with low sensitivity to B_1_^+^ by Marques et al.^1^ (C) The protocol optimized for FLAWS contrast by Beaumont et al.^11^ (Table 2) to demonstrate the achieved contrasts

The clinically feasible jointly optimized UNI and FLAWS protocol is summarized in Table 2, and the CNR curves are given in Supporting Information Figure S5. The FA combination of 4/5° was chosen instead of 4/4° to improve GM–CSF CNR in the UNI image (an example using 4/4° is given in Supporting Information Figure S4). For this protocol and 2 other protocols (Table 2), simulated UNI signal intensities for a 50%–150% B_1_^+^ range versus T_1_ are plotted in Supporting Information Figure S6, and images with the UNI bias fields are shown in Figure 5. It is important to note that this protocol has a higher resolution (0.65 × 0.65 × 0.65 vs. 1 × 1 × 1 and 0.8 × 0.8 × 0.8 mm^3^) and shorter TR_MP2RAGE_ (4 vs. 8 and 5 s), and hence a shorter acquisition time (7:18 vs. 12:50 and 10:02 min:s) compared to the other 2. Figure 6 demonstrates the contrast in 3 common areas (40 × 40 mm^2^) with different B_1_^+^ values.

**FIGURE 7:**
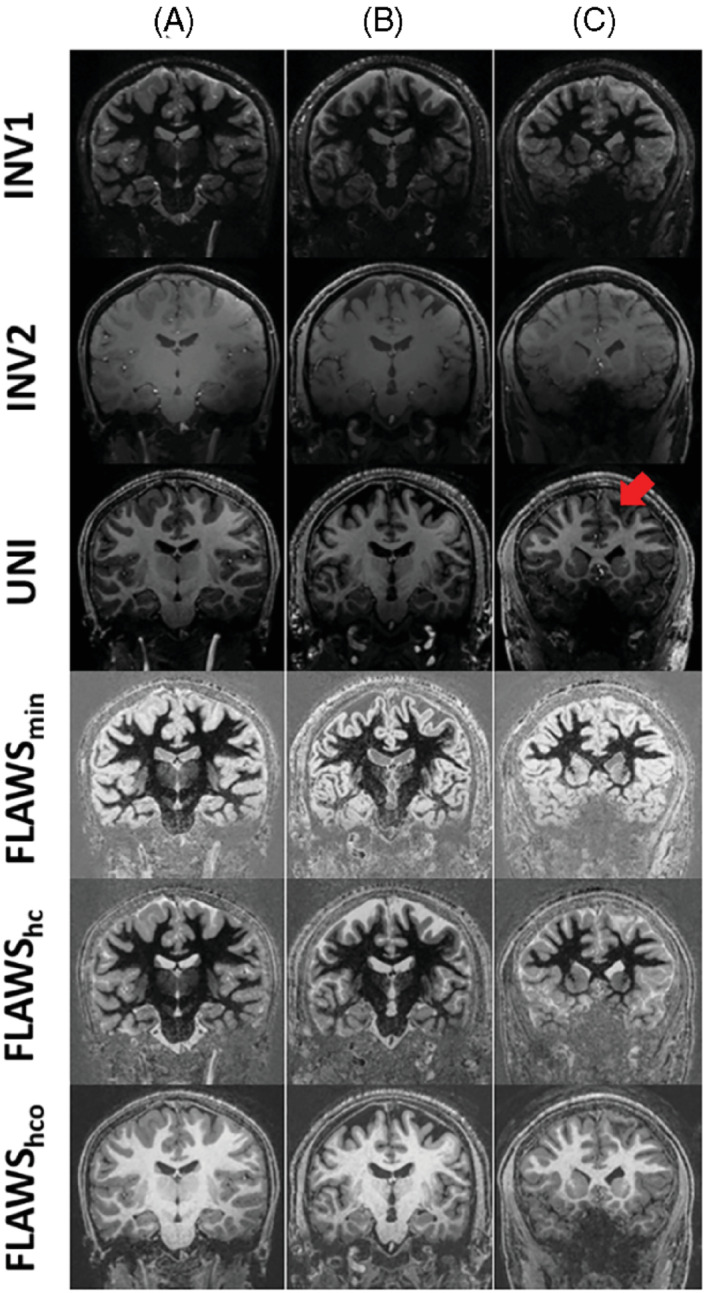
Coronal images with different contrasts acquired at TR_MP2RAGE_ = 4000 ms from (A) 10‐year‐old healthy child, (B) 62‐year‐old healthy adult, and (C) 12‐year‐old pediatric epilepsy patient with focal cortical dysplasia. The lesion is visible in the left hemisphere (e.g., as a hypo‐intense region indicated by the red arrow in the WM of the UNI image). The nominal image resolution was 0.65 mm isotropic. The scan duration (min:s) was 7:18 for pediatric subjects and 8:58 for the adult subject.

Using this protocol, images were obtained in 17 subjects aged 10–62 years. Figure 7 exemplifies the high‐quality images obtained from 10‐year‐old and 62‐year‐old healthy subjects and from a pediatric epilepsy patient with focal cortical dysplasia. The epileptogenic lesion is visible in all patient images and can easily be identified as a hypointensity in left hemisphere WM in the UNI image.

## 4 DISCUSSION AND CONCLUSION

In this study, a single MP2RAGE acquisition was optimized using EPG simulations to obtain high‐resolution (0.65 mm isotropic) FLAWS and MP2RAGE UNI images at 7T while accounting for B_1_^+^ variabilities. It was possible to obtain high‐contrast FLAWS_min_, FLAWS_hc_, and FLAWS_hco_ images while maintaining at least 85% of the maximum UNI image CNR. Not only were these images acquired within 1 acquisition eliminating the need for coregistration,^4^ but also the short scan duration (7:18 min:s) makes it feasible for use in clinical populations. This enabled us to obtain pediatric scans both in healthy children and children with epilepsy, important as MP2RAGE with resolution <0.8 mm isotropic is a recommended sequence for imaging epilepsy at 7T.^12^

Optimization of the MP2RAGE protocol was made in 2 steps consisting of TI optimization followed by FA optimization. This process was performed at 3 different TR_MP2RAGE_s that were constrained by the requirement for high‐resolution and short‐scan duration. Across all TR_MP2RAGE_s, shorter TI_1_ durations provided higher total CNR for the simultaneous optimization of FLAWS and MP2RAGE UNI. This was in agreement with the results from the original FLAWS study,^4^ where shorter TI_1_ durations resulted in better WM suppression. TI_1_ minimization is challenging, particularly in the context of high‐resolution protocols with large matrix sizes. A partial Fourier factor of 6/8 enabled shorter TI_1_ values in practice^4^ while allowing for high‐resolution due to earlier sampling of the k‐space center compared to fully sampled acquisitions. Different TR_GRE_ values have not been explored systematically in this study; however, larger bandwidths and hence shorter TR_GRE_ values are expected to result in reduced SNR, whereas longer TR_GRE_ values further limit the ability to use more optimal short TI_1_ values. Results from 1 subject demonstrated higher SNR with the narrower bandwidth (BW = 160 Hz/Px) without visually apparent chemical shift artifacts.

Another goal of these simulations was to compare the maximum possible CNR values for each TR_MP2RAGE_ (and therefore a range of scan duration). By reducing the TR_MP2RAGE_ from 5000 to 4000 ms, the CNR per unit time was expected to be approximately 20% lower. To provide high‐resolution images in a relatively short clinically applicable scan duration, a GRAPPA factor of 4 was required in the second PE direction, together with a TR_MP2RAGE_ value of 4000 ms. Some reduction in image quality compared to the GRAPPA factor 3 scans was visible, consistent with lower overall SNR (partially mitigated by the self‐calibrating acquisition with 40 lines of fully sampled k‐space center) and some localized image quality degradation related to local increases in the geometry factor.^29^, ^30^ Nevertheless, good‐quality images were acquired in children as exemplified in Figure 7. Another solution to reduce the scan time would be the compressed sensing (CS) approaches.^31^, ^32^, ^33^, ^34^ Trotier et al. applied CS to the MP2RAGE sequence in mice at 7T^35^ using a variable density Poisson disk trajectory.^34^, ^35^, ^36^ Similarly, Mussard et al. used CS‐MP2RAGE at 3T in healthy subjects using a trajectory based on a jittered Cartesian spiral phyllotaxis pattern.^37^, ^38^, ^39^ Trotier et al. were able to obtain images in healthy humans at 3T with 0.8 mm isotropic resolution in 6:21 (min:s) using CS‐MP2RAGE.^40^ Whereas the CS approaches are very promising to reduce the scan time, optimizing the parameters both on the acquisition and reconstruction side is challenging.^39^ In this work, the focus was on contrast optimization using the standard MP2RAGE sequence with simple linear phase‐encode ordering. It would be possible to combine our work with more flexible encoding schemes, such as CS, which could enable greater acceleration.

### 4.1 Methodological considerations

One potential limitation of these (and related Bloch^10^, ^11^) simulations was their sensitivity to the particular T_1_ values used for WM and GM, which shifted the optimum values of FAs in the WM–GM CNR plots by approximately ±1°. Because T_1_ values change with age,^41^ and a range of T_1_ values is always encountered in a single subject's brain, this limitation is unavoidable. Nevertheless, excellent anatomical detail could be achieved across the age range of 10–62 using the final protocol, which is at least in part due to the optimization over a wide range of B_1_^+^ factors. Similarly to the Bloch simulations, if the number of phase‐encoding steps changes, a new optimum combination of FAs might be needed; however, this can be achieved in approximately 20 min utilizing the parallel computing feature of MATLAB (R2018a, The Mathworks, Natick, MA).

One key feature of the MP2RAGE sequence is its capacity to generate T_1_ maps.^1^ This is potentially compromised in protocols designed to produce FLAWS contrast, where low GM signal in the UNI image (used to derive T_1_) leads to poor T_1_ estimates. In one study, synthetic image contrasts (e.g., synFLAWS) were instead generated after data acquisition using MP2RAGE T_1_ maps.^42^ Although powerful, this requires some further data postprocessing. Here, multiple contrasts were generated within a single scan. An important future development would be to understand how the current protocol performs in terms of T_1_ map generation, including the effect of reducing TR_MP2RAGE_. T_1_ map generation using this protocol would enable both direct and synthetic contrasts.

Some small oscillations were present in the EPG simulations when T_2_ values and diffusion were taken into account, which implies that EPG simulations considering all effects should be slightly more accurate if the PSF of the signal is considered.

### 4.2 Comparison to previous MP2RAGE optimizations

FLAWS and MP2RAGE UNI images have been typically optimized separately. In the original MP2RAGE study,^1^ CNR was optimized for the UNI image. Smaller values of *α*_1_ were suggested to restrict the range of the signal intensities such that the B_1_^+^ variability would be managed to some extent at the expense of reduced resolution and CNR.^1^, ^11^ In this study, the simulations covered a large B_1_^+^ range (50%–140%), and CNR plots resulted from utilizing these different results for each B_1_^+^. This made the optimizations relevant in the context of large B_1_^+^ variability without imposing any FA restrictions. Although the simulations (Supporting Information Figure S6) implied that our protocol could be more sensitive to variations in B_1_^+^ compared to the low‐B_1_^+^‐sensitive MP2RAGE protocol, this was not visually apparent (Figures 5 and 6). Signal intensities for different tissue T_1_s decrease monotonically at different B_1_^+^, maintaining relative local contrast between tissues despite the possibility of low–spatial frequency contrast variation.

In the study by Beaumont et al.,^11^ which optimized the MP2RAGE sequence for FLAWS contrast, the UNI images had a GM‐suppressed contrast. To compensate for this, new ratio combinations were proposed, such as the FLAWS_hco_ to provide T_1_‐weighted image contrast.^11^ Here, in 7 min 18 s using a shorter TR_MP2RAGE_ of 4000 ms and higher resolution (0.65 mm), we provided an image protocol able to produce UNI images with typical contrast facilitating comparison with most current MP2RAGE UNI images by the use of existing processing pipelines tailored to this contrast. Added value is gained by producing high‐quality FLAWS_min/hc/hco_ images in addition to a UNI image. With a view to future clinical application, the use of the method by O'Brien et al.^28^ was applied to obtain a full contrast set with image background suppression, thereby providing a complimentary range of contrasts from a single scan that were demonstrated to provide good visualization of pathology in a pediatric epilepsy scan.

MP2RAGE scans are sensitive to motion. Even though our scan was just over 7 min, this is still a long period to remain motionless for some clinical populations, for example, children. For instance, motion artifact was observable in the INV2 image of the pediatric patient in Figure 7. This motivates the implementation of motion compensation (real‐time or retrospective) in future work. Although effective motion correction^43^ might enable longer TR_MP2RAGE_ times and correspondingly scan durations, CNR gains are likely to be modest, and motion correction remains challenging at 7T owing to concomitant changes in static B_0_ ^44^ and B_1_^−^ fields^45^ with head pose. A larger sample size is also needed to verify which images (FLAWS_min_, FLAWS_hc_, FLAWS_hco_, and MP2RAGE UNI) provide contrast improvement and confer clinical utility.

## FUNDING INFORMATION

This research was supported by Great Ormond Street Hospital Children's Charity (GOSHCC) Sparks Grant V4419, King's Health Partners, in part by the Medical Research Council (UK) (grants MR/K006355/1 and MR/LO11530/1) and Medical Research Council Center for Neurodevelopmental Disorders, King's College London (MR/N026063/1), and by core funding from the Wellcome Engineering and Physical Sciences Research Council Centre for Medical Engineering at King's College London [WT203148/Z/16/Z]. j.o.m., k.v., and c.c. were funded by a Sir Henry Dale Fellowship jointly by the Wellcome Trust and the Royal Society (206675/Z/17/Z). c.c. was also funded by a grant from GOSHCC (VC1421). r.m. was funded by the Innovate UK grant (68539). Infrastructure support was provided by the National Institute for Health Research Mental Health Biomedical Research Centre at South London, Maudsley NHS Foundation Trust, King's College London and the National Institute for Health Research Mental Health Biomedical Research Centre at Guy's and St Thomas' Hospitals NHS Foundation Trust. This research was funded in whole, or in part, by the Wellcome Trust [WT203148/Z/16/Z and 206675/Z/17/Z]. For the purpose of open access, the author has applied a CC BY public copyright licence to any Author Accepted Manuscript version arising from this submission.

## CONFLICT OF INTEREST

Raphael Tomi‐Tricot is an employee at Siemens Healthineers, and Ronald Mooiweer is seconded to Siemens Healthineers.

## Supporting information


**VIDEO S1:** The UNI and FLAWS_min_ CNR plots using different FA combinations at a fixed TI_1_/TI_2_ of 650/2220 ms and TR_MP2RAG_ of 4000 ms. The plots represent the simulation results for 10 different B_1_^+^ values (50% B_1_^+^ to 140% B_1_^+^ with steps of 10%). The upper rows show the CNRs for the UNI image between WM‐GM and GM‐CSF, and the total CNR. The lower rows show the CNRs for the FLAWS_min_ image between the GM‐WM and GM‐CSF, and the total CNR

**FIGURE S1:** The UNI images acquired using two different *α*_2_ values while keeping other scan parameters the same from four healthy subjects demonstrating the GM‐CSF contrast reversal, in line with the simulation results shown in Figure 3. The contrast reversal is easily observed in the caudate nuclei and lateral ventricles indicated by the red and blue rectangles
**FIGURE S2:** Simulated UNI signal intensities for WM, GM, and CSF for TI_1_/TI_2_ = 650/2280 ms and TR_MP2RAGE_ = 4000 ms for an FA range of 1–10 ° to investigate the effect of the inversion pulse efficiency (*eff*). The *eff* values of 0.96 and 1 were tested with subtle differences that did not affect the CNR optimizations
**FIGURE S3:** RF Spoiling, Diffusion, and T_2_ effects on the simulated UNI signals. 0 means the effect was not considered and 1 means it was included in the simulations. TI_1_/TI_2_ of 650/2280 ms, TR_MP2RAGE_ of 4000 ms, and *α*_1_/*α*_2_ = 4/5 ° were used. The curves generated overlapped when the T_2_ effects were not included in the simulations. The case which included all effects was very similar to these curves with additional small oscillations
**FIGURE S4:** UNI images acquired using (A) BW = 350 Hz/Px, *α*_1_/*α*_2_ = 3/3 ° TI1/TI2/TR_MP2RAGE_ = 650/2220/4000 ms (B) the same parameters as in A except *α*_1_/*α*_2_ = 3/4° which should improve the UNI GM‐CSF contrast, (C) BW = 160 Hz/Px, *α*_1_/*α*_2_ = 4/4 ° TI_1_/TI_2_/TR_MP2RAGE_ = 650/2220/4000 ms. The only difference between C and the final protocol is that *α*_2_ = 5° in the final protocol which gives an improved UNI GM‐CSF contrast
**FIGURE S5:** The UNI and FLAWS_min_ CNR plots using different FA combinations at a fixed TI_1_/TI_2_ of 650/2280 ms and TR_MP2RAGE_ of 4000 ms which led to the final protocol. The plots represent the average of simulation results using 10 different B_1_^+^ values (50% B_1_^+^ to 140% B_1_^+^ with steps of 10%). The upper row shows the CNRs for the UNI image between WM‐GM and GM‐CSF, and the total CNR. The lower row shows the CNRs for the FLAWS_min_ image between the GM‐WM and GM‐CSF, and the total CNR. The FA combination of *α*_1_/*α*_2_ = 4/4 ° results in a better FLAWS_min_ GM‐CSF contrast but worse UNI GM‐CSF contrast compared to *α*_1_/*α*_2_ = 4/5°, which was the combination chosen for the final protocol
**FIGURE S6:** UNI Signal Intensity simulated for different T_1_ values using three different protocols our final protocol (Table 2), low‐B_1_^+^‐sensitive MP2RAGE protocol^1^ and the FLAWS protocol.^11^ The protocols have different TR_MP2RAGE_ values and nominal resolutions as indicated in the legend. The dashed curves correspond to the signal intensities simulated using 50% and 150% B_1_^+^ while the solid curves are the signal intensities at 100% B_1_^+^

## Data Availability

The codes for the simulations can be downloaded from https://github.com/mriphysics/EPG‐X and https://github.com/siladokumaci/MP2RAGE_EPG_simulations. The clinical datasets that support the findings of this study are available on request from the corresponding author, Ayşe Sıla Dokumacı. The data are not publicly available due to containing information that could compromise the privacy of research participants.
