# Supplementary material for: Simultaneous Optimization of MP2RAGE T1 ‐weighted (UNI) and FLuid And White matter Suppression (FLAWS) brain images at 7T using Extended Phase Graph (EPG) Simulations
Source: Magn Reson Med. 2022 Nov 9;89(3):937–50. doi: 10.1002/mrm.29479 (PMC10100108; doi:10.1002/mrm.29479)
Supplement: Supplementary file 2 — FIGURE S1: The UNI images acquired using two different α 2 values while keeping other scan parameters the same from four healthy subjects demonstrating the GM‐CSF contrast reversal, in line with the simulation results shown in Figure 3. The contrast reversal is easily observed in the caudate nuclei and lateral ventricles indicated by the red and blue rectangles FIGURE S2: Simulated UNI signal intensities for WM, GM, and CSF for TI1/TI2 = 650/2280 ms and TRMP2RAGE = 4000 ms for an FA range of 1–10 ° to investigate the effect of the inversion pulse efficiency (eff). The eff values of 0.96 and 1 were tested with subtle differences that did not affect the CNR optimizations FIGURE S3: RF Spoiling, Diffusion, and T2 effects on the simulated UNI signals. 0 means the effect was not considered and 1 means it was included in the simulations. TI1/TI2 of 650/2280 ms, TRMP2RAGE of 4000 ms, and α 1/α 2 = 4/5 ° were used. The curves generated overlapped when the T2 effects were not included in the simulations. The case which included all effects was very similar to these curves with additional small oscillations FIGURE S4: UNI images acquired using (A) BW = 350 Hz/Px, α 1/α 2 = 3/3 ° TI1/TI2/TRMP2RAGE = 650/2220/4000 ms (B) the same parameters as in A except α 1/α 2 = 3/4° which should improve the UNI GM‐CSF contrast, (C) BW = 160 Hz/Px, α 1/α 2 = 4/4 ° TI1/TI2/TRMP2RAGE = 650/2220/4000 ms. The only difference between C and the final protocol is that α 2 = 5° in the final protocol which gives an improved UNI GM‐CSF contrast FIGURE S5: The UNI and FLAWSmin CNR plots using different FA combinations at a fixed TI1/TI2 of 650/2280 ms and TRMP2RAGE of 4000 ms which led to the final protocol. The plots represent the average of simulation results using 10 different B1 + values (50% B1 + to 140% B1 + with steps of 10%). The upper row shows the CNRs for the UNI image between WM‐GM and GM‐CSF, and the total CNR. The lower row shows the CNRs for the FLAWSmin image between the GM‐WM and GM‐CS [file MRM-89-937-s002.docx]

**SUPPORTING INFORMATION FIGURES**


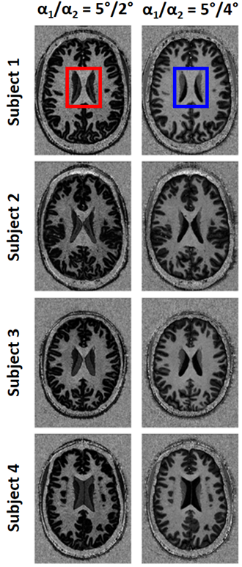


**Fig. S1**. The UNI images acquired using two different α_2_ values while keeping other scan parameters the same from four healthy subjects demonstrating the GM-CSF contrast reversal, in line with the simulation results shown in Fig. 3. The contrast reversal is easily observed in the caudate nuclei and lateral ventricles indicated by the red and blue rectangles.


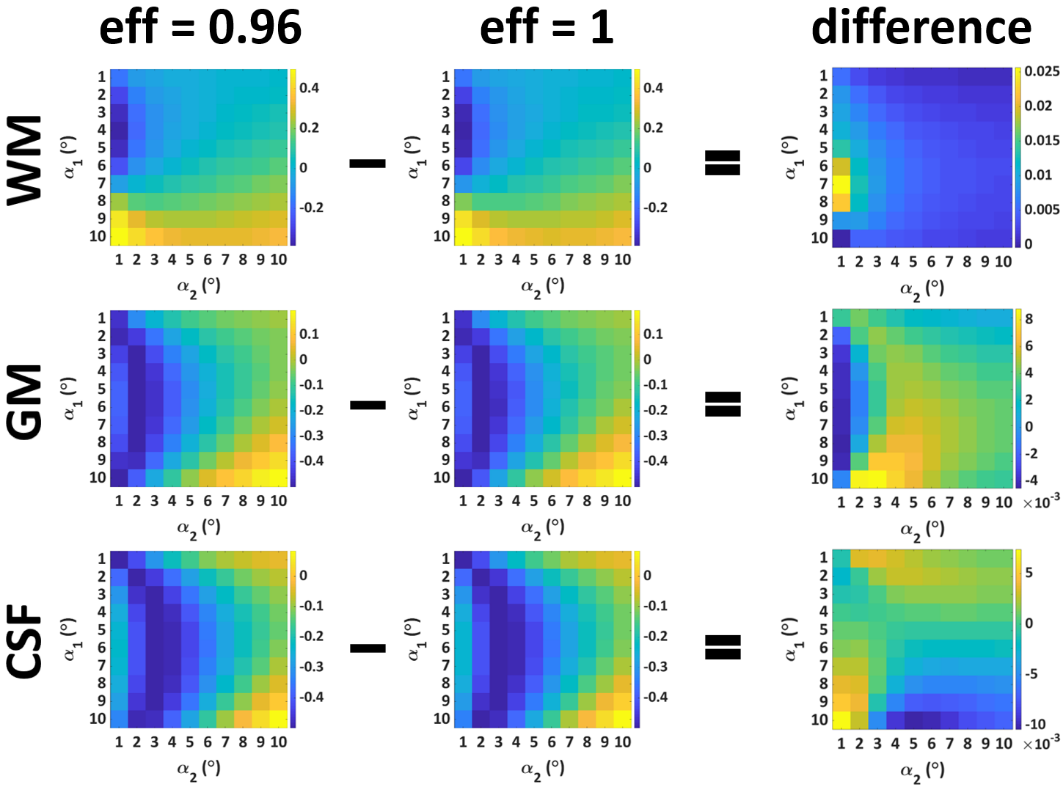


**Fig. S2.** Simulated UNI signal intensities for WM, GM, and CSF for TI1/TI2 = 650ms/2280ms and TR_MP2RAGE_ = 4000 ms for an FA range of 1° to 10° to investigate the effect of the inversion pulse efficiency (*eff*). The *eff* values of 0.96 and 1 were tested with subtle differences that did not affect the CNR optimisations.


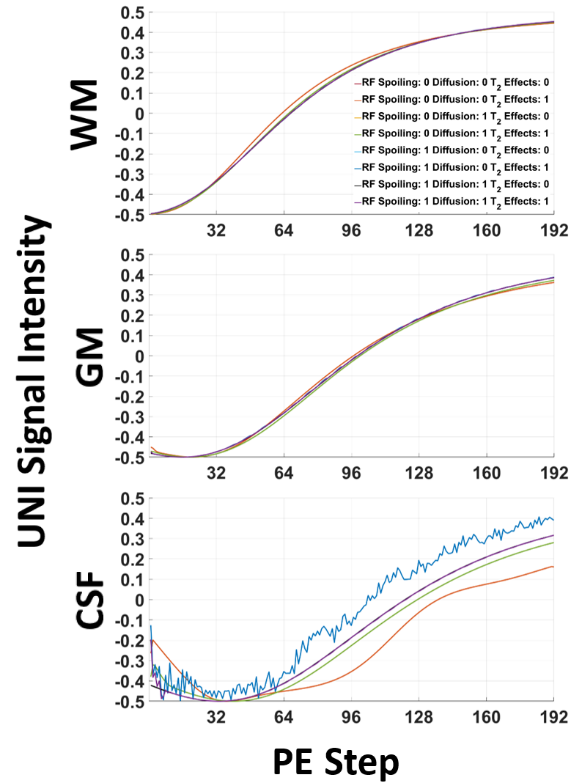


**Fig. S3.** RF Spoiling, Diffusion, and T_2_ effects on the simulated UNI signals. 0 means the effect was not considered and 1 means it was included in the simulations. TI_1_/TI_2_ of 650ms/2280ms, TR_MP2RAGE_ of 4000 ms, and α_1_/α_2_ = 4°/5° were used. The curves generated overlapped when the T_2_ effects were not included in the simulations. The case which included all effects was very similar to these curves with additional small oscillations.


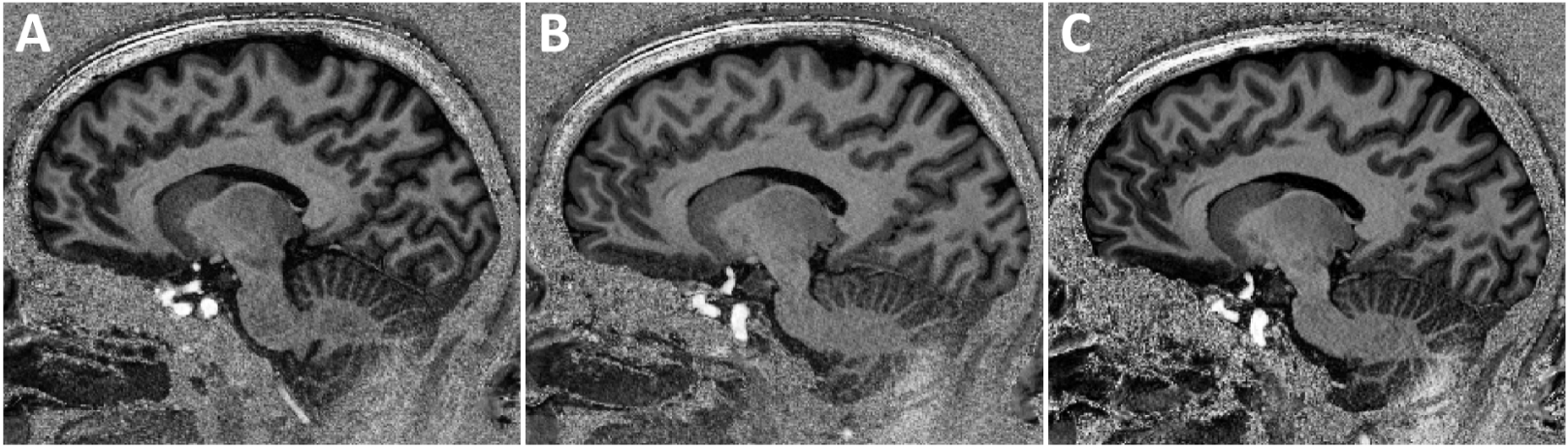
**Fig. S4.** UNI images acquired using A) BW = 350Hz/Px, α_1_/α_2_ = 3°/3° TI1/TI2/TR_MP2RAGE_ = 650ms/2220ms/4000ms B) the same parameters as in A except α_1_/α_2_ = 3°/4° which should improve the UNI GM-CSF contrast, C) BW = 160Hz/Px, α_1_/α_2_ = 4°/4° TI1/TI2/TR_MP2RAGE_ = 650ms/2220ms/4000ms. The only difference between C and the final protocol is that α_2_ = 5° in the final protocol which gives an improved UNI GM-CSF contrast.


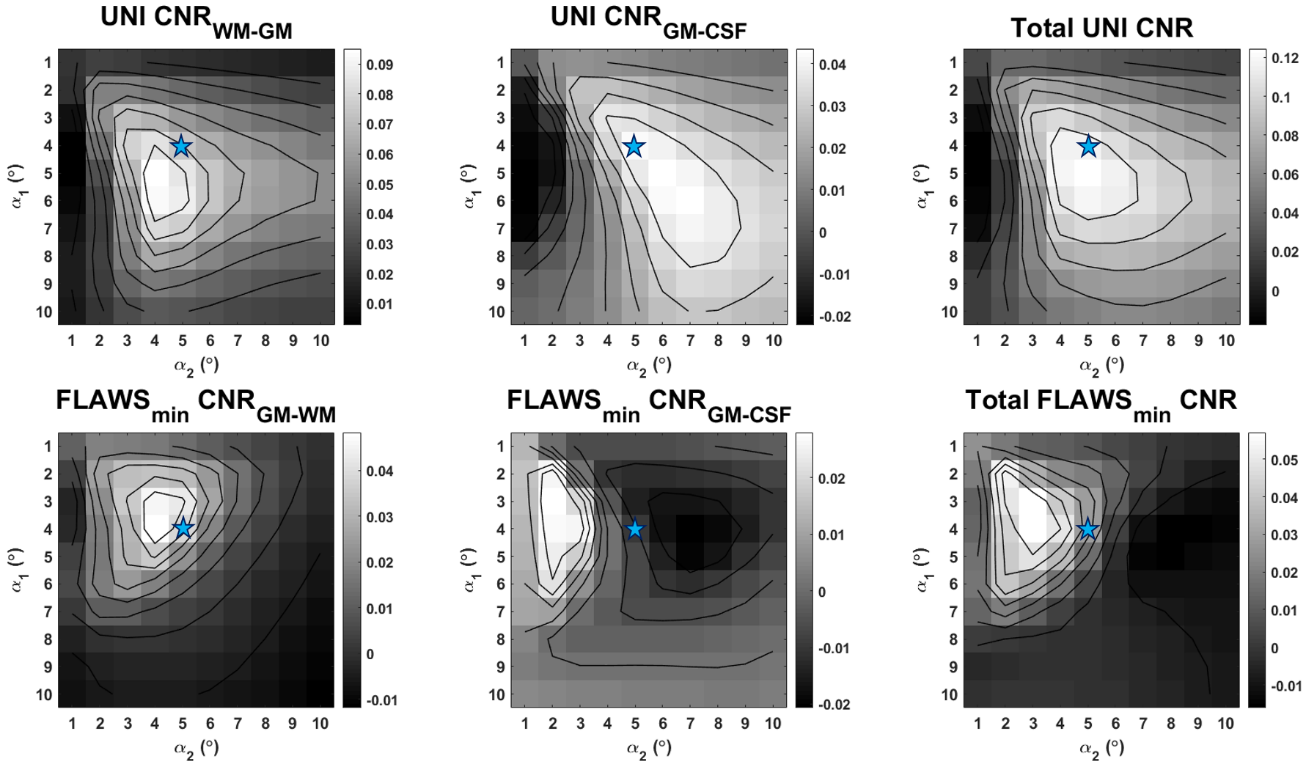


**Fig. S5.** The UNI and FLAWS_min_ CNR plots using different FA combinations at a fixed TI_1_/TI_2_ of 650ms/2280ms and TR_MP2RAGE_ of 4000 ms which led to the final protocol. The plots represent the average of simulation results using 10 different B_1_^+^ values (50% B_1_^+^ to 140% B_1_^+^ with steps of 10%). The upper row shows the CNRs for the UNI image between WM-GM and GM-CSF, and the total CNR. The lower row shows the CNRs for the FLAWS_min_ image between the GM-WM and GM-CSF, and the total CNR. The FA combination of α_1_/α_2_ = 4°/4° results in a better FLAWS_min_ GM-CSF contrast but worse UNI GM-CSF contrast compared to α_1_/α_2_ = 4°/5°, which was the combination chosen for the final protocol.


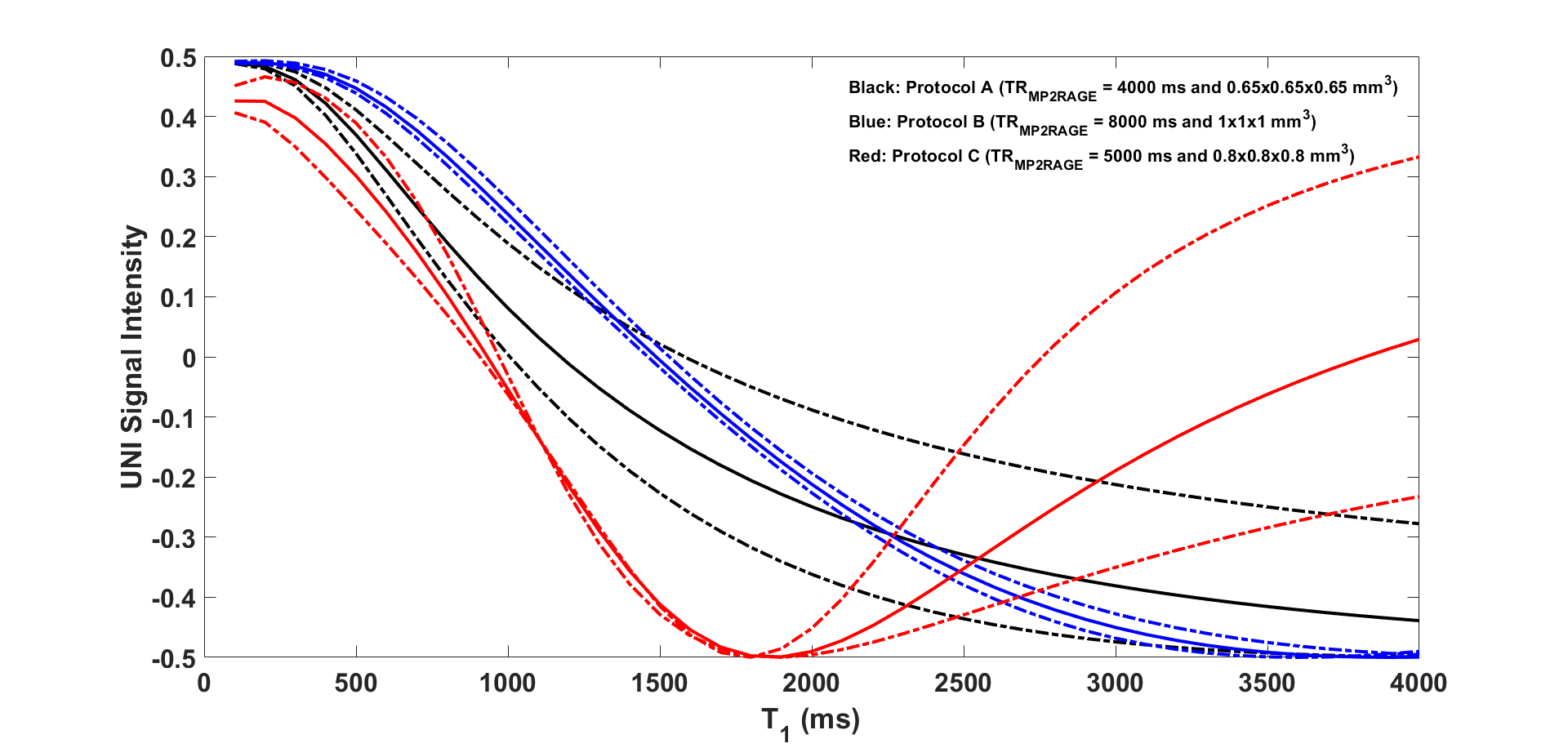
**Fig. S6.** UNI Signal Intensity simulated for different T_1_ values using three different protocols (our final protocol (Table 1b), low-B_1_^+^-sensitive MP2RAGE protocol (1) and the FLAWS protocol (11). The protocols have different TR_MP2RAGE_ values and nominal resolutions as indicated in the legend. The dashed curves correspond to the signal intensities simulated using 50% and 150% B_1_^+^ while the solid curves are the signal intensities at 100% B_1_^+^.
